# Action of Opium on the Genito-Urinary Organs

**Published:** 1861-09

**Authors:** B. Woodward

**Affiliations:** Galesburg, Illinois


ARTICLE III.

### ACTION OF OPIUM ON THE GENITO-URINARY ORGANS.

By B. WOODWARD, M. D., Galesburg, Ill.

Though opium has been known as a therapeutic agent from the earliest ages, it is not yet fully understood; and as the relations of pathology and therapeutics are being studied, new forms of its action are being developed. Almost all writers on *Materia Medica* unite in saying, "that opium arrests all the secretions, except those of the skin." This is the general belief; and I had taken it for granted, till about three years ago, when an accident made me doubt the assertion, so far as the action was concerned, and subsequent experience has convinced me, that under certain circumstances, instead of arresting, it increases this secretion in a remarkable manner. Having occasion to take a dose of morphine, I was struck with the largely increased urinary secretion, and its clear, limpid character. In order to test the matter, I next day measured all the urine passed in 24 hours, and proved it to be ℥xxviii, specific gravity 1014. The next day after urinating, on rising from bed, I ate and drank as usual, and took a third of a grain of sulph. morphia, at 7, 10, 1 and 4 o'clock, and measured all the urine passed at 9 p. m., and found it to ℥ xlv, very limpid, specific gravity 1003. This experiment was repeated carefully four times, at intervals of five days, and each time proved a corresponding increase in quality, and lowering of specific gravity. Since that time I have several times repeated the experiment, and always with the same results. Lest this should be the result of some idiosyncrasy, I subjected five young men to the experiment, and with four of them, obtained a very large increase, and lower specific gravity. With one of them, on two trials, I found no perceptible increase or diminution of the quantity, but a marked lessening of specific gravity. It is proper to say, that I have not obtained the same results with opium itself, as with its alkaloids, but there has been no difference, whether the muriate or sulph. morphia were used. Acting on these hints, I have repeatedly used morphine in irritable conditions of the nervous system where a diuretic was required, and have always been pleased with the results. These are the class of cases requiring a sedative action, and in which veratrum viridi acts on the kidneys. Opium then, appears to be a sedative diuretic, causing an increased secretion from the kidneys, by its relaxing properties. I think it will be found that in many cases of disease, the urinary secretion is arrested by the state of nervous tension, which has been superinduced, and that instead of a resort to stimulant diuretics, sedatives will relax the tension, and allow the secretion to be restored. This sedative action must be brought about by such agents as shall act on and through the nervous system, and not those which act on the blood, (*e. g.* calomel or antimony.) There is a vital difference between sedation of the nervous system and depression. Depression is often caused by nervous irritation, which wears out the powers, and which is successfully combatted by sedatives, which allay the irritation and give the system a chance to recuperate. Whether by the use of opium the solid contents of the urine are increased, could only be determined by an analysis of all passed in a given time; but the evidence of my own is, that they are not. Where there is evidence of a poisoning of the system by retention of excrementitious matter which should be eliminated by the kidneys, I have found the better plan to be, to combine morphine with a saline diuretic. Retention of excrementitious matters, produces nervous irritation, and this leads to true depression. Another marked action of opium is as an anaphrodisiac. For obvious reasons it is difficult to settle this satisfactorily, but in the cases of several women whom I knew to be opium eaters, inquiry of the husbands has elicited the fact, that in them, the sexual desire was almost extinct; and several men, of whom I have inqnired, who used much opium, have acknowledged the same to be true of them. This may account for the impunity, so far as health is concerned, with which Turks and other Asiatics, who all use much opium, keep large numbers of women in their harems. Their lives are spent in a dreamy voluptuousness, while, in fact, sexual appetite may not be largely indulged. The testimony of travelers is, "that large families of children are rare among the wealthy orientals who keep extensive harems." The testimony of a prostitute on this point was, "that she was obliged to use opium freely, so that she should be merely passive, while admitting men to her embrace, or she should have been worn out." I could give the cases of several men for whom I have prescribed opium, to enable them to overcome their lustful propensities, and always with benefit, as it held the desire in abeyance, and enabled them to bring their moral powers to bear. In the case of a most estimable woman, now dead, who, ten days after accouchment, became the victim of uncontrolable sexual desire, amounting, almost, to nymphomania, full doses of morphine by the mouth, and solutions of morphine to the parts, acted almost like a charm, and restored her to herself.

I conclude then that opium has a direct action on the nerves, governing the urinary and generative organs.

